# Modelization of the regulation of protein synthesis following fertilization in sea urchin shows requirement of two processes: a destabilization of eIF4E:4E-BP complex and a great stimulation of the 4E-BP-degradation mechanism, both rapamycin-sensitive

**DOI:** 10.3389/fgene.2014.00117

**Published:** 2014-05-06

**Authors:** Sébastien Laurent, Adrien Richard, Odile Mulner-Lorillon, Julia Morales, Didier Flament, Virginie Glippa, Jérémie Bourdon, Pauline Gosselin, Anne Siegel, Patrick Cormier, Robert Bellé

**Affiliations:** ^1^Ifremer, UMR6197, Laboratoire de Microbiologie des Environnements ExtrêmesPlouzané, France; ^2^Université de Nice-Sophia Antipolis, UMR 7271, Laboratoire I3SSophia, Antipolis, France; ^3^Sorbonne Universités, UPMC University Paris 06, UMR 8227, Integrative Biology of Marine Models, Translation Cell Cycle and DevelopmentStation Biologique de Roscoff, Roscoff cedex, France; ^4^CNRS, UMR 8227, Integrative Biology of Marine Models, Translation Cell Cycle and DevelopmentStation Biologique de Roscoff, Roscoff cedex, France; ^5^CNRS UMR 6241, Laboratoire LINA, Université de NantesNantes, France; ^6^CNRS, IRISA-UMR 6074, Campus de BeaulieuRennes, France; ^7^INRIA, Centre Rennes – Bretagne Atlantique, Symbiose, Campus de BeaulieuRennes, France

**Keywords:** translational control, sea urchin embryos, mechanisms of fertilization, deterministic model, translation simulation

## Abstract

Fertilization of sea urchin eggs involves an increase in protein synthesis associated with a decrease in the amount of the translation initiation inhibitor 4E-BP. A highly simple reaction model for the regulation of protein synthesis was built and was used to simulate the physiological changes in the total 4E-BP amount observed during time after fertilization. Our study evidenced that two changes occurring at fertilization are necessary to fit with experimental data. The first change was an 8-fold increase in the dissociation parameter (k_off1_) of the eIF4E:4E-BP complex. The second was an important 32.5-fold activation of the degradation mechanism of the protein 4E-BP. Additionally, the changes in both processes should occur in 5 min time interval post-fertilization. To validate the model, we checked that the kinetic of the predicted 4.2-fold increase of eIF4E:eIF4G complex concentration at fertilization matched the increase of protein synthesis experimentally observed after fertilization (6.6-fold, *SD* = 2.3, *n* = 8). The minimal model was also used to simulate changes observed after fertilization in the presence of rapamycin, a FRAP/mTOR inhibitor. The model showed that the eIF4E:4E-BP complex destabilization was impacted and surprisingly, that the mechanism of 4E-BP degradation was also strongly affected, therefore suggesting that both processes are controlled by the protein kinase FRAP/mTOR.

## Introduction

Early development of the sea urchin embryo is one among the models which contribute to establish the paradigms of the mechanisms of translation, the eukaryotic universal process for protein biosynthesis (Mathews et al., 2007). Many factors are involved in all steps of translation machinery, namely initiation, elongation, and termination and have been extensively reviewed (Mathews et al., 2007). For the great majority of the mRNAs, the initiation step of translation occurs on activated mRNAs that contain a m^7^GpppN molecule (where N is any of the four nucleotides) at the 5' end, also known as a cap structure, thus referred as cap-dependent translation initiation (Jackson et al., 1995). Not exhaustively, protein translation initiation begins with the binding of the eukaryotic initiation factor 4E (eIF4E) to the cap-structure of the mRNA. The protein eIF4E recruits a large scaffolding protein called eIF4G that interacts, among others, with eIF4A and eIF3 linking the 5' end of the mRNA and the 43S preinitiation complex (Gingras et al., 1999a). After scanning from the 5' end to the start codon, the first amino acid is incorporated and the peptide elongation step proceeds (Jackson et al., 2010, 2012; Hinnebusch, 2011). The protein eIF4E is a major target for regulation of translation initiation (Sonenberg and Gingras, 1998). Its availability in the cells depends on the presence of 4E-BPs, eIF4E binding proteins, which compete with eIF4G for a common binding site on eIF4E (Mader et al., 1995). Thus 4E-BPs sequester eIF4E, and consequently prevent cap-dependent translation (Haghighat et al., 1995). The 4E-BPs phosphorylation status regulates their interaction with eIF4E: underphosphorylated 4E-BPs bind to eIF4E and inhibit cap-dependent translation, whereas hyperphosphorylated forms do not (Pause et al., 1994). Most of the data available to date indicate that FRAP/mTOR (FKBP12 and Rapamycin-Associated Protein/mammalian Target Of Rapamycin) is the main kinase that phosphorylates 4E-BP (Brunn et al., 1997; Burnett et al., 1998) on four conserved residues in metazoans (Gingras et al., 1999b).

Among the experimental advantages of the sea urchin embryo model, genes encoding all factors involved in translation are present and are non-redundant; thus, while mammalian genomes contain three 4E-BP homologs, sea urchin contains an unique form (Morales et al., 2006). At fertilization, an increase in protein synthesis occurs, which is necessary for the initiation of development and for the occurrence of the first cell cycle (Epel, 1990; Cormier et al., 2003; Gilbert, 2003). The protein synthesis increase is independent of new transcription and involves cap-dependent initiation from maternal mRNAs already present in the unfertilized egg (Cormier et al., 2003). Fertilization triggers 4E-BP release from eIF4E and consequently protein synthesis translation through a rapamycin-, and thus, mTOR-sensitive pathway (Cormier et al., 2001; Salaun et al., 2003, 2004). An original mechanism of 4E-BP regulation first demonstrated in sea urchin, corresponds to the rapid disappearance of 4E-BP pool following fertilization (Salaun et al., 2003, 2004, 2005; Oulhen et al., 2007, 2009). Although fertilization is known to provoke many changes in the egg metabolism that could interfere on translation regulation (Epel, 1990; Gilbert, 2003; Parrington et al., 2007), we demonstrate here that a reduced model involving 4E-BP, eIF4E, and eIF4G is sufficient to explain the changes observed at fertilization on 4E-BP level and on the increase of cap-dependent translation. Simulations using this minimal model suggest that both a 32.5-fold increase of the 4E-BP degradation mechanism and an 8-fold change in the dissociation constant (k_off1_) of eIF4E:4E-BP complex are required at fertilization and should occur in a 5 min time interval after fertilization. Furthermore, simulations of changes after fertilization in the presence of rapamycin show that the drug strongly affects the eIF4E:4E-BP complex stability and, unexpectedly, the 4E-BP degradation mechanism implying that both mechanisms are controlled by the FRAP/mTOR protein kinase.

## Materials and methods

### Protein production and purification

The construction of the plasmid pAr encoding eIF4E1 from *Mus musculus* has already been described (Pyronnet et al., 1999) and the protocol to produce and purify Flag-tagged *Mm*IF4E1 was performed as described herein. The cloning of *Strongylocentrotus purpuratus* 4E-BP and the production of the recombinant His-tagged *Sp*4E-BP protein was performed as described in Gosselin et al. (2011). Briefly, recombinant *Sp*4E-BP was produced in BL21 (DE3) strain *E.coli* (Novagen) and purified on an affinity column, packed with a chelating sepharose Fast Flow Resin preloaded with Ni^2+^ ions (Amersham Pharmacia Biotech). A second purification step was performed on a Superdex 75 column (Amersham Pharmacia Biotech). After concentration with Amicon® Ultra-4 Centrifugal Filter Units (10 kDa), concentration of the purified proteins was estimated with a Nanodrop ND-1000.

Purification of native eIF4E was performed from *Sphaerechinus granularis* egg extracts using m^7^GTP beads as described previously (Oulhen et al., 2010). After washing, beads were suspended in Laemmli loading buffer. The amount of recovered *Sg*IF4E was determined after resolution on SDS-PAGE electrophoresis and revelation by Coomassie blue staining. Quantification was performed by comparison with a range of BSA concentrations migrated on the same gel. The bands were quantified using the ImageJ 1.43j program (Wayne Rasband, National Institutes of Health, USA) after digitization of the stained gel.

### Preparation of gametes and fertilization

*Sphaerechinus granularis* sea urchins collected in the Brest area (France) were maintained in running seawater. Spawning of gametes, fertilization, and cell culture were as described (Oulhen et al., 2010). When indicated, 20 μM rapamycin (LC laboratories) from a 20 mM stock solution in ethanol was added to the eggs 20 min before fertilization. Cleavage was scored under a light microscope. Each experiment used gametes from a single female exhibiting greater than 90% fertilization.

### Quantification of 4E-BP and eIF4E in sea urchin eggs

To quantify eIF4E and 4E-BP in the sea urchin unfertilized eggs, total crude protein extracts were prepared by direct dissolution of 20 μl egg pellet (corresponding to 12,000 eggs) with 150 μl of SDS-Fix buffer as described (Belle et al., 2011). A range of different extract volumes was submitted to SDS-PAGE and Western-blotted together with known amounts of purified *Sg*IF4E or recombinant *Sp*4E-BP as prepared above. Proteins in the different samples were immuno-revealed using mouse monoclonal antibody directed against eIF4E from rabbit (BD transduction laboratories, Lexington, KY) or rabbit polyclonal antibodies directed against human 4E-BP2 (Rousseau et al., 1996), a generous gift from Nahum Sonenberg (McGill University, Montreal, Quebec, Canada). The antigen-antibody complex was measured by chemiluminescence using horseradish peroxydase-coupled secondary antibodies according to the manufacturer's instructions (ECL or ECL+, GE Healthcare Life Sciences). Quantification of the bands was done using the ImageJ 1.43j program. Measurement has been performed on extracts from eggs of 9 different females for eIF4E and 4 females for 4E-BP. Protein concentrations were calculated considering an egg volume of 0.3 nl (diameter 80 μM).

### Determination of protein synthesis *in vivo*

Unfertilized eggs (5% suspension in sea water) were incubated in the presence of [^35^S]- L- methionine during 1 h at the final concentration of 10 μCi/ml. When indicated, 20 μM rapamycin was added to the incubation mixture 20 min prior to fertilization. Eggs were then harvested by centrifugation, rinsed three times, re-suspended in Millipore-filtered sea water in the presence or absence of rapamycin and fertilized. At different times, batches (500 μl) of the embryo suspension were taken; the cells were pelleted and frozen in liquid nitrogen. Soluble protein extracts were prepared as described in Oulhen et al. (2010). [^35^S]- L-methionine incorporation into proteins was measured on duplicate aliquots of the extracts after 10% TCA precipitation on Whatman 3 M filters and counting in a scintillation counter in the presence of Optiphase Supermix scintillation liquid.

### Measurement of 4E-BP level after fertilization

At different times, following fertilization, 20 μl pelleted embryos were dissolved in 150 μl of SDS-Fix buffer to prepare total crude protein extracts as above. Proteins were separated on SDS-PAGE and 4E-BP was analyzed by immunoblotting using the rabbit polyclonal antibodies directed against human 4E-BP (see above). The antigen-antibody complex was revealed by chemoluminescence, the signal was digitalized and quantified using the ImageJ 1.43j software. Densitometric analysis gave relative numerical values, which were used to calculate percentages of 4E-BP present in embryos considering as a reference (i.e., 100%) the quantity of total 4E-BP in unfertilized eggs. When necessary, percents were converted into absolute concentrations based on values for 4E-BP calculated as described above.

### Surface plasmon resonance (SPR) data acquisition

Data were acquired with Reichert SR7000DC spectrometer instrument (Buffalo, USA). Running buffer was HEPES 50 mM pH 7.7 KCl 150 mM 0.5% Igepal and flow rate was 25 μL.min^−1^. 60 μRIU of *Sp*4E-BP was immobilized on a mixed SAM (1 C_11_-(OEG)_6_-COOH:10 C_11_-(OEG)_3_-OH) (Buffalo, USA) *via* classical amine coupling chemistry. For interaction experiment, concentration of Flag-*Mm*IF4E1:m^7^GTP was assayed by spectrophotometry and stock solution was centrifuged before use. Then a concentration range from 4.37 μM to 53.9 nM of Flag-*Mm*IF4E1:m^7^GTP (3-fold dilution series) was injected on the *Sp*4E-BP chip at 25°C. Following each Flag-*Mm*IF4E1:m^7^GTP injection, the chip was regenerated with 30 s of 10 mM NaOH. Each curve displayed was double referenced with a set of 6 blank buffer injections. Data were then fitted using a global analysis method with Scrubber 2.0a software (Biologic Software, Australia). Fitting errors are reported for each kinetic constant.

## Results

### The minimal model elaboration

A model for cap-dependent translation initiation in sea urchin has been proposed (Belle et al., 2010) based on the systemic environment of BIOCHAM software (Calzone et al., 2006). The Biocham model contains too many reactions and undetermined parameters to allow quantitative simulations of dynamic changes occurring at fertilization. We therefore investigated the strategy of extracting a minimal core model. The model was constructed with the following reactions: (R1) the reversible association between eIF4E and 4E-BP, (R2) the reversible association of eIF4E to eIF4G, (R3) the irreversible production of protein by the initiation complex containing associated eIF4E:eIF4G, (R4) the synthesis of 4E-BP by the initiation complex containing associated eIF4E:eIF4G and (R5) the degradation of free 4E-BP.

(R1) eIF4E + 4E-BP <−> eIF4E:4E-BP (k_on1_/k_off1_ = 1/KD_1_)
(R2) eIF4E + eIF4G <−> eIF4E:eIF4G (k_on2_/k_off2_ = 1/KD_2_)
(R3) eIF4E:eIF4G -> Protein + eIF4E:eIF4G (k_catProtein_)
(R4) eIF4E:eIF4G -> 4E-BP + eIF4E:eIF4G (k_cat4E−BP_)
(R5) 4E-BP -> 0 (k_lys4E−BP_)

In this model, eIF4E is considered to be always associated to mRNAs since its affinity for the cap structure and the stability of the eIF4E: cap mRNA complex were demonstrated to be very high in other species (Sonenberg and Gingras, 1998). Global protein production is assumed to be proportional to the amount of eIF4E:eIF4G complex (R4). The total amounts of eIF4E and eIF4G were shown to remain constant after fertilization in sea urchin (Oulhen et al., 2007).

The differential equations resulting from the model using the law of mass action are as follow:

1. d[eIF4E]/dt = k_off1_[eIF4E:4E-BP] + k_off2_[eIF4E:eIF4G] − k_on1_[eIF4E][4E-BP] − k_on2_[eIF4E][eIF4G]
2. d[4E-BP]/dt = k_off1_[eIF4E:4E-BP] + k_cat4E−BP_[eIF4E:eIF4G] − k_lysis4E−BP_[4E-BP] − k_on1_[eIF4E][4E-BP]
3. d[eIF4G]/dt = k_off2_[eIF4E:eIF4G] − k_on2_[eIF4E][eIF4G]
4. d[eIF4E:4E-BP]/dt = k_on1_[eIF4E][4E-BP] − k_off1_[eIF4E:4E-BP]
5. d[eIF4E:eIF4G]/dt = k_on2_[eIF4E][eIF4G] − k_off2_[eIF4E:eIF4G]
6. d[Protein]/dt = k_catProtein_[eIF4E:eIF4G]

### Parameter determination in unfertilized eggs

The relevance of the core model in unfertilized eggs was studied by combining experimental data acquisition and learning procedure of unknown parameters. Initial total concentrations of 4E-BP, eIF4E, and eIF4G as well as kinetic parameters of the association of eIF4E:4E-BP and eIF4G:eIF4E complexes had to be experimentally determined and were further used to fit parameters for 4E-BP synthesis and degradation.

#### Concentrations of the translation factors

The concentrations of 4E-BP and eIF4E in unfertilized eggs were determined by immunoblotting and densitometric quantification as indicated in the Materials and Methods section. The total concentration of 4E-BP and eIF4E were respectively 3.67 μM (*SD* = 0.83, *n* = 4), and 2.15 μM (*SD* = 0.29, *n* = 6) thus indicating an excess of total 4E-BP over total eIF4E. The concentration of eIF4G could not be determined using the procedure described for 4E-BP and eIF4E for two reasons: first, sea urchin eIF4G was present under complex post-translational modified forms (Oulhen et al., 2007) and it was therefore not clear which bands could be ascribed to eIF4G, and second, no significant and measurable amounts of endogenous nor recombinant protein had been obtained. However, it was previously demonstrated that after fertilization a rapid exchange of 4E-BP for eIF4G occurred on eIF4E with a stoichiometric behavior (Oulhen et al., 2007). Therefore, simulations were performed using a minimal eIF4G concentration equivalent to eIF4E concentration 2.15 μM.

#### Determination of the kinetic parameters

**Figure 1:**
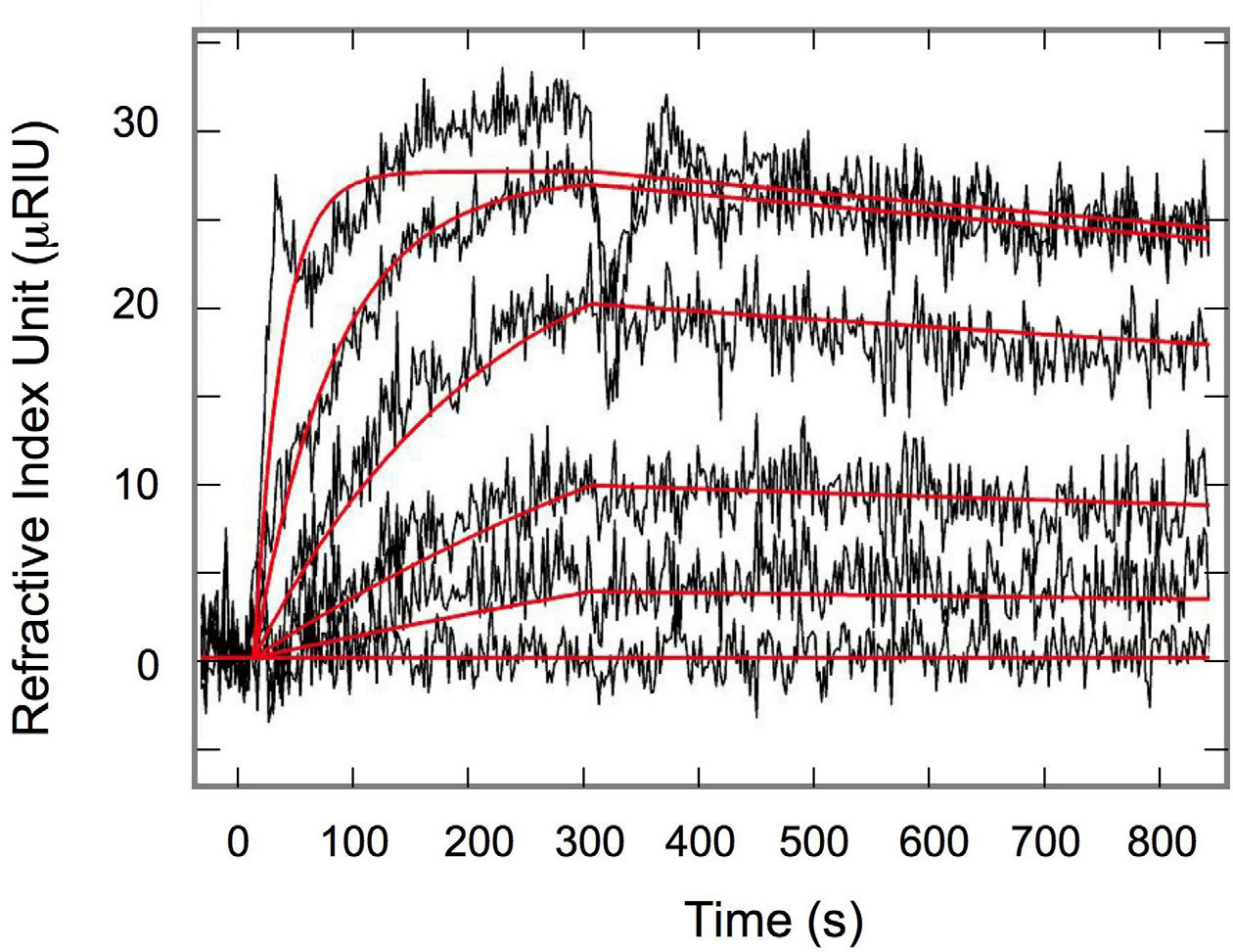
**Surface plasmon resonance Interaction of eIF4E and 4E-BP**. Each sensorgram, relative refractive index (μRIU) as a function of time, was obtained after injection of eIF4E from 4.37 μM to 53.9 nM according to a three-fold dilution range. Overlapped signals were fitted using a single equilibrium kinetic isotherm (solid red lines).

The kinetic parameters for 4E-BP: eIF4E interaction were determined by surface plasmon resonance (SPR). 4E-BP was immobilized on a chip and several concentrations of eIF4E were injected onto the chip. Figure 1 shows the overlapped sensorgrams in relative Index of Refraction Unit (RIU) and their associated theoretical fits (solid lines) resulting from global data analysis according to a one step 1:1 reaction. The kinetic parameters determined were k_on1_ = 9.3 ± 0.1 × 10^3^ M^−1^.s^−1^, k_off1_ = 2.2 ± 0.1 ×10^−4^ s^−1^ and the affinity constant *KD*_1_ = 23 nM (Table 1), all in good concordance with published data in other species (Abiko et al., 2007; Mizuno et al., 2008). Regarding the high conservation of the binding sites among species (Joshi et al., 2005) the measured parameters likely reflect the values in the sea urchin *Sphaerechinus granularis*.

**Table 1:** **Parameters of the model for translation changes at fertilization (see text for details)**.

	**Unfertilized eggs**	**Fertilized eggs**	**Fertilized eggs + rapamycin**	**Method of determination**
k_on1_	9.3 × 10^3^ M^−1^ s^−1^			SPR
k_off1_	2.2 × 10^−4^ s^−1^	×8	×1	SPR and simulation
KD_1_	23 nM			
k_on2_	1.82 × 10^3^ M^−1^ s^−1^			SPR-Bibliography
k_off2_	2.0 × 10^−4^ s^−1^			SPR-Bibliography
KD_2_	110 nM			
k_lys4E-BP_	5.9 × 10^−4^ s^−1^	×32.5	×16	Emetine treatment of eggs and simulation
k_cat4E-BP_	3.2 × 10^−3^ s^−1^			Simulation
Time interval of kinetic parameter change		5 min	33 min	Simulation
4E-BP_total_	3.67 μM			Immunoblots densitometry
eIF4E _total_	2.15 μM			Immunoblots densitometry
eIF4G_total_	2.15 μM			Bibliography/Estimation
4E-BP	1.90 μM	×0.11		Simulation/model perturbation
eIF4E	0.02 μM	×12.9		Simulation/model perturbation
eIF4G	1.79 μM	×0.36		Simulation/model perturbation
eIF4E:4E-BP	1.77 μM	×0.20		Simulation/model perturbation
eIF4E:eIF4G	0.36 μM	×4.2	×2.4	Simulation/model perturbation
Protein synthesis	1	×6.6	×3.5	Radioactive rate of methionine incorporation into proteins

A direct measurement of eIF4E:eIF4G interaction could not be analyzed by surface plasmon resonance. A k_on2_ = 1.82 × 10^3^ M^−1^.s^−1^ and a k_off2_ = 2.0 × 10^−4^ s^−1^ for eIF4E:eIF4G interaction were used (Table 1) from published data in other species (Von Der Haar et al., 2006; Umenaga et al., 2011), resulting in determining an affinity constant for *KD*_2_ = 110 nM (Table 1).

#### Parameters for 4E-BP synthesis and degradation

**Figure 2:**
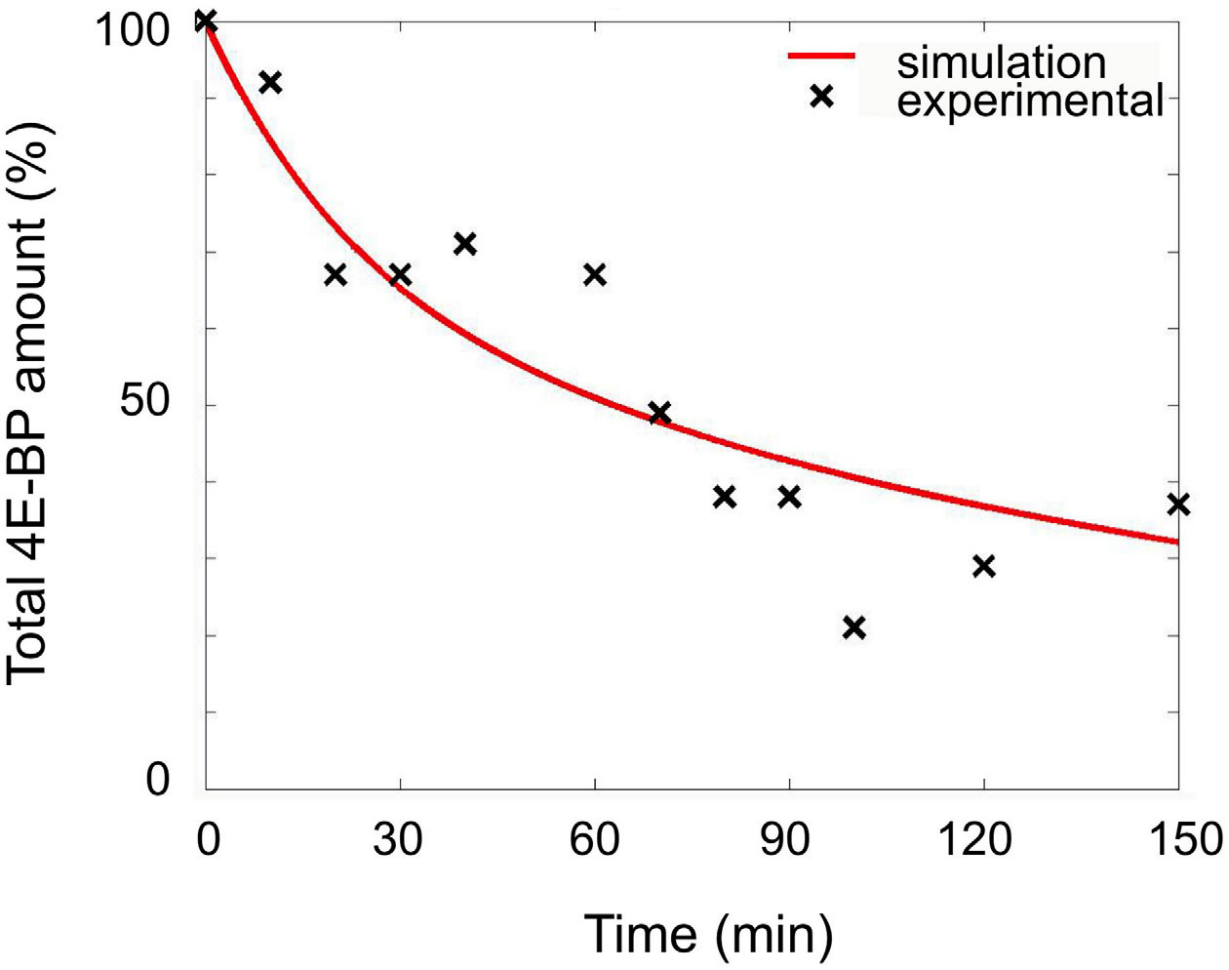
**Determination of the degradation rate of 4E-BP in unfertilized eggs**. Unfertilized eggs were incubated in the presence of 100 μM emetine. The amount of total 4E-BP remaining upon time was determined from immunoblotting experiments as indicated in the material and methods section. The values from 8 independent experiments are plotted as black cross. Simulation using the model (reactions R1, R2, and R5) leading to the best fit is shown as red line. The total amount of 4E-BP is expressed as % of initial value.

The constants for the synthesis of 4E-BP (k_cat4E−BP_) and its degradation (k_lys4E−BP_) had to be determined. The degradation constant of reaction R5 was calculated from experiments consisting in the measurement of the total amount of 4E-BP remaining after treatment of unfertilized eggs in the presence of the protein synthesis inhibitor emetine. In that condition, the disappearance of 4E-BP depends on reactions R1, R2, and R5 since no production could occur (R4). The disappearance of total 4E-BP was simulated and the best fitting value for k_lys4E−BP_ was determined by optimizing the least square error to the experimental values (Figure 2). The best fit was obtained for a value of 5.9 × 10^−4^ s^−1^ (Table 1).

Since 4E-BP remains constant in the untreated unfertilized egg, the synthesis constant, k_cat4E−BP_ (R4), could be calculated using reactions R1 to R5. A least-square method was used to constraint a constant value, as close as possible to 100%, for the total amount of 4E-BP over time; the inferred catalytic constant for 4E-BP synthesis was k_cat4E−BP_ = 3.2 × 10^−3^ s^−1^ (Table 1).

#### Calculation of factor concentrations

The unfertilized eggs are in a steady state in which the total concentrations of 4E-BP, eIF4E, and eIF4G remain constant as verified from immunoblotting experiments over the period of conservation of the eggs. Using reactions R1 and R2, the concentrations of free and complexed forms of 4E-BP, eIF4E, and eIF4G could be calculated. From the differential reactions (4) and (5) of the model:

(4) d[eIF4E:4E-BP]/dt = k_on1_[eIF4E][4E-BP] − k_off1_[eIF4E:4E-BP] = 0
(5) d[eIF4E:eIF4G]dt = k_on2_[eIF4E][eIF4G] − k_off2_[eIF4E:eIF4G] = 0,

and knowing the total concentrations:

[4E-BP]_total_ = 3.67 μM, [eIF4E]_total_ = [eIF4G]_total_ = 2.15 μM, the two equations become:
k_on1_([eIF4E]total−[eIF4E:4E-BP]−[eIF4E:eIF4G]) ([4E-BP]total−[eIF4E:4E-BP])−k_off1_[eIF4E:4E-BP] = 0
k_on2_([eIF4E]total−[eIF4E:4E-BP]−[eIF4E:eIF4G]) ([eIF4G]total−[eIF4E:eIF4G])−k_off2_[eIF4E:eIF4G] = 0

from which the unknown values [eIF4E:4E-BP] and [eIF4E:eIF4G] can be calculated. From these, the free amounts of [eIF4E], [4E-BP],and [eIF4G]are deduced from the total amount minus the complexed forms

[eIF4E] = [eIF4E]_total_ − [eIF4E:4E-BP]- [eIF4E:eIF4G]
[4E-BP] = [4E-BP] _total_ − [eIF4E:4E-BP]
[eIF4G] = [eIF4G] _total_ − [eIF4E:eIF4G]

The calculated values are all reported in Table 1.

At that point, the parameters and steady state concentrations of all the reactions of the model are known in the unfertilized eggs (Table 1). Since protein synthesis was determined by [^35^S]-methionine incorporation into all translated proteins and expressed as a percentage of incorporation, the k_catProtein_ could not be determined. However, since in the model, the amount of protein synthesized is proportional to the concentration of [eIF4E:eIF4G], changes in [eIF4E:eIF4G] will reflect the changes in the protein synthesis rates.

### Learning parameters and concentrations after fertilization

**Figure 3:**
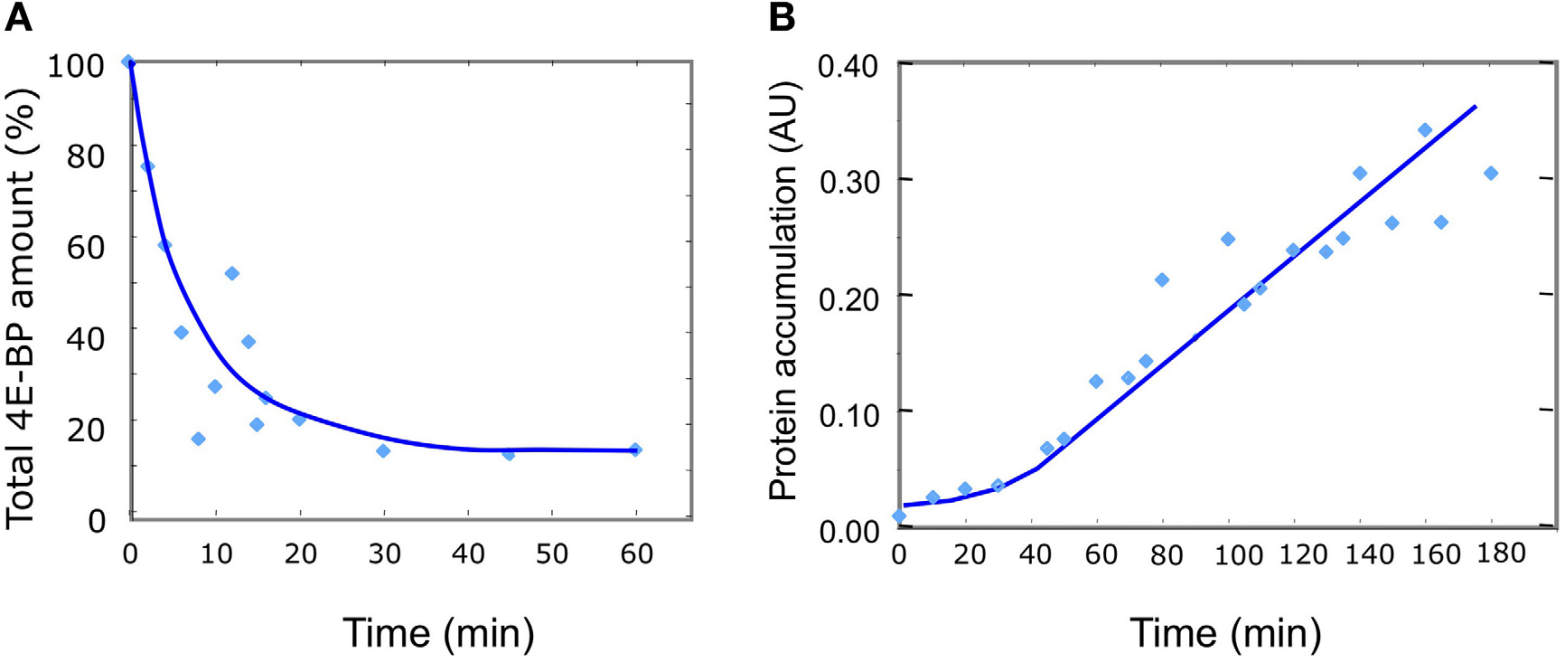
**Changes in 4E-BP total amount and protein synthesis *in vivo* after fertilization. (A)** 4E-BP was measured by immunoblotting and densitometric quantification as described in the material and methods section. Mean values from 10 independent experiments are plotted as a function of time. The total amount of 4E-BP is expressed as % of initial value. **(B)** Protein synthesis was determined from incorporation into proteins of [^35^S]methionine at different times after fertilization as indicated in the Materials and Methods section. Mean values of protein accumulation from 11 independent determinations are plotted as a function of time in arbitrary unit (UA).

The kinetic of 4E-BP disappearance observed *in vivo* reveals two main features: a decrease of the total 4E-BP protein starting from fertilization and lasting about 15 min, followed by a stabilization of the level of the protein at 18–20% of the initial amount (Figure 3A). In parallel, the rate of protein synthesis increases and stabilizes around 30 min after fertilization resulting in the linear accumulation of neosynthetized proteins (Figure 3B). Therefore, fertilization corresponds to a time-dependent change from a steady state (unfertilized state) to another one (fertilized state) reached after 30 min. With respect to the minimal model proposed, two non-exclusive changes could explain the experimental values of total 4E-BP evolution: a destabilization of eIF4E:4E-BP complex (R1) and an increase in the degradation rate k_lysis4E−BP_ of 4E-BP. The two hypotheses were simulated separately. Since the biochemical modifications induced by fertilization are not instantaneous (Epel, 1990; Cormier et al., 2003; Gilbert, 2003), the time necessary for the biochemical modifications to occur at fertilization through the transduction pathway was introduced in the model as a parameter time change varying linearly from the initial value of parameters in unfertilized eggs to their final value after fertilization.

**Figure 4:**
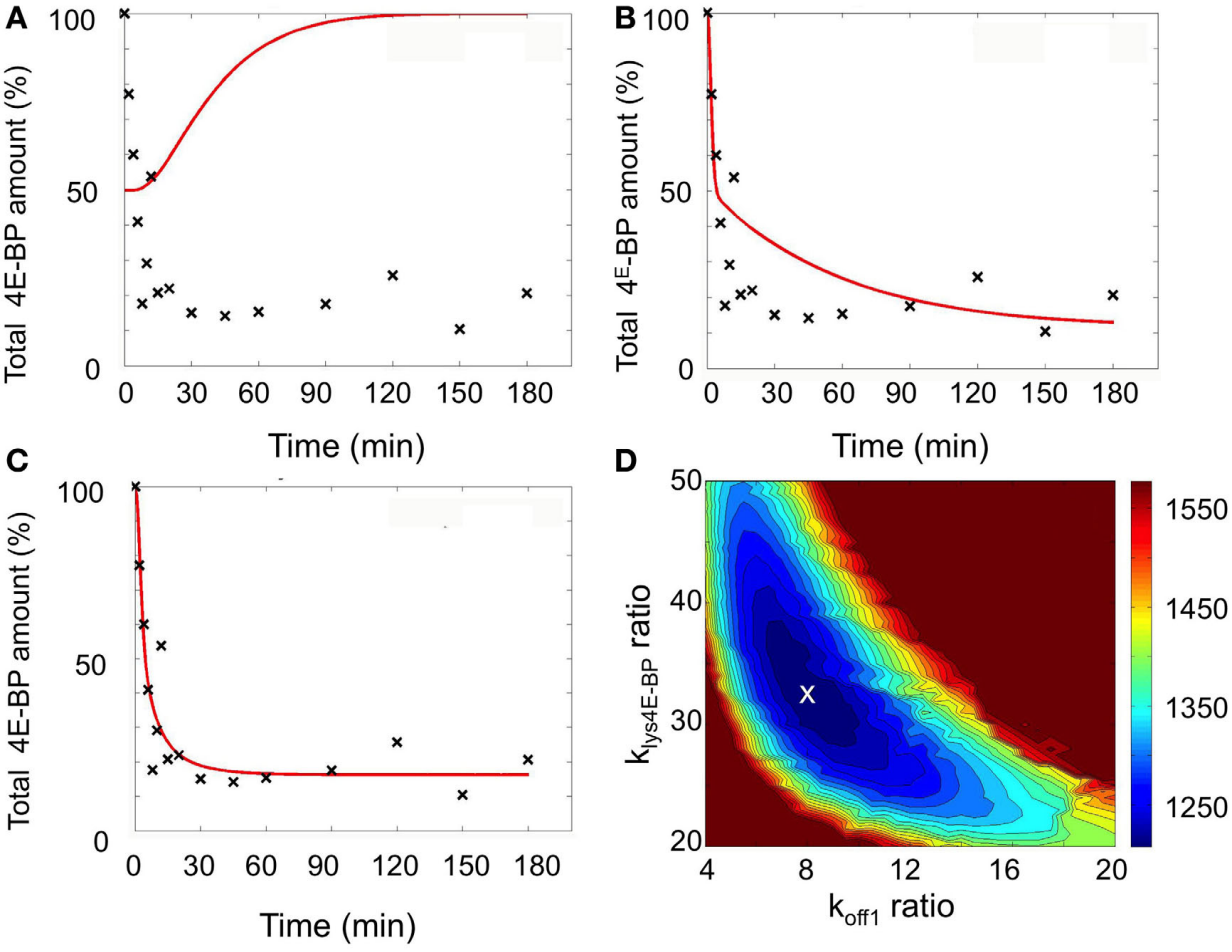
**Simulation of fertilization changes on 4E-BP using the minimal model**. Best fits (red curves) compared to experimental data (black cross) are shown: **(A)** Ranging k_off1_ from unfertilized value (1 to 100-fold) and the parameter time change (1 to 15 min). **(B)** Ranging k_lys4E-BP_ from unfertilized value (1–100-fold) and the parameter time change (1–15 min). **(C)** Changing k_off1_ and k_lys4E-BP_ from unfertilized value (1–100-fold) and the parameter time change (1–15 min). **(D)** Color representation of the fit according to the change of k_off1_ and k_lys4E-BP_ when the parameter time change is fixed to 5 min (k_cat4E-BP_ and k_off1_ ratio are the ratio between the value reached after fertilization and the unfertilized value). The color scale on the right was associated to distance to data (sum of square residual) obtained in the simulations. The white cross corresponds to the best fit. The total amount of 4E-BP is expressed as % of initial value.

A set of model simulations was first performed by perturbing the value k_off1_ (R1) ranging from its unfertilized egg value (Table 1) up to 100 times the value and by introducing a parameter time change ranging between 1 and 15 min. For each simulation, the mean-square error between the model prediction and 4E-BP experimental data was computed. The least-worst fit (sum of square residual = 46712) is shown in Figure 4A and, not surprisingly, the simulation led to a non-biologically relevant increase in the total amount of 4E-BP. A second set of simulations was performed by perturbing the value of k_lysis4E−BP_ranging from its value in unfertilized eggs (Table 1) to 100 times the value while still introducing a parameter time change ranging from 1 and 15 min. The least-worst fit (sum of square residual = 3122) is shown in Figure 4B and the corresponding simulation did not fully match the experimental data.

Therefore, simulations were performed by perturbing both the k_off1_ value (R1), and the k_lysis4E−BP_ (R4)values together with a parameter time change ranging from 1 to 15 min. Using least square method, a very good fit with the experimental data was obtained (sum of square residual = 1207). The best fit was obtained when k_off1_ increased 8-fold from the value in unfertilized eggs and k_lysis4E−BP_ increased 32.5-fold with a parameter time change of 5 min (Figure 4C). Figure 4D shows in a color scale the least square sum of square residual values obtained in the simulations for the ranging values of k_off1_ and k_lysis4E−BP_. The best fit (white cross) was located in a small area related to the accuracy of the determinations.

**Figure 5:**
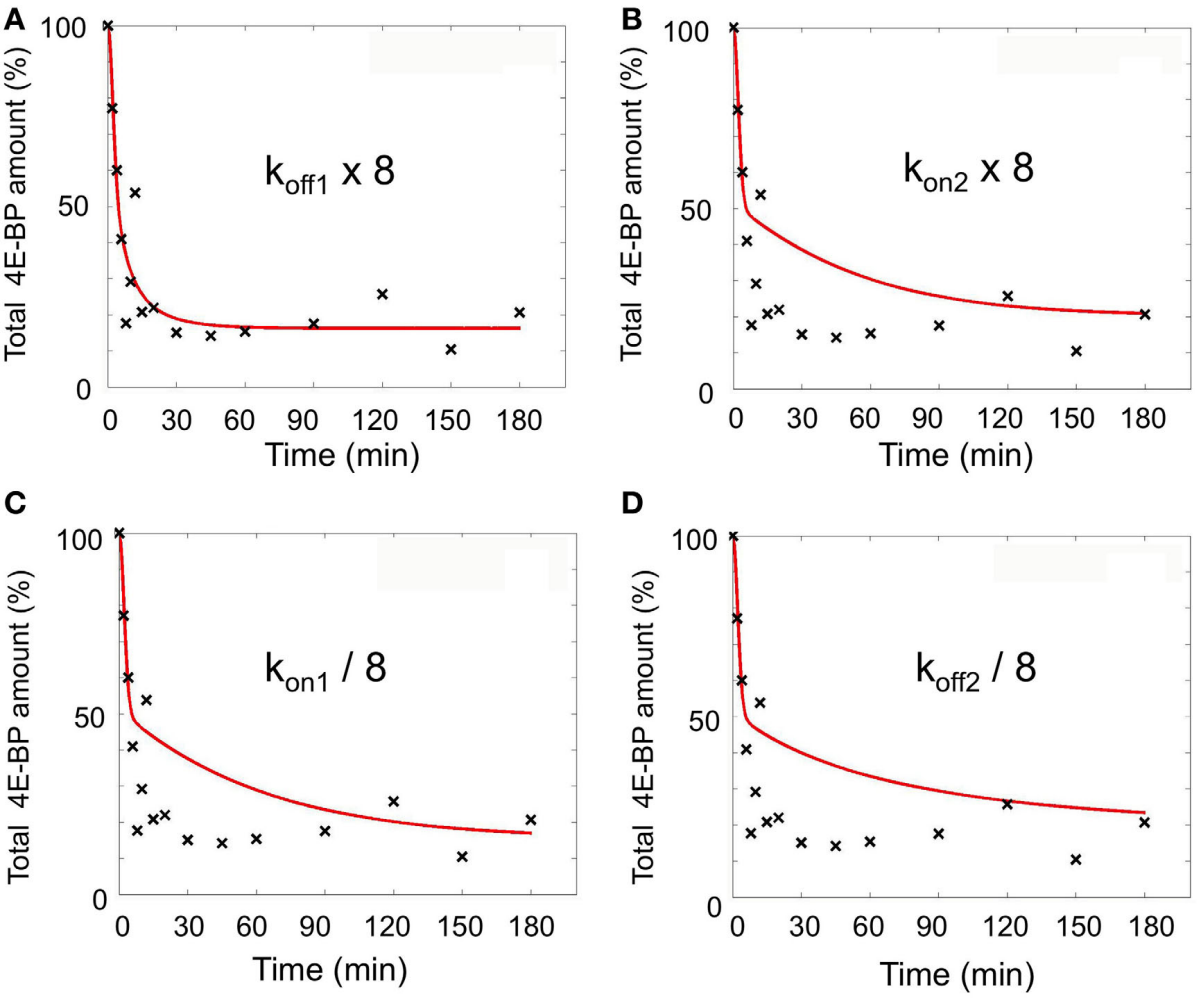
**Simulations performed varying the indicated parameters (k_off1_, k_on2_, k_on1_, k_off2_) at fertilization**. k_lys4E-BP_ was fixed as 32.5-fold increase compared to unfertilized eggs. Parameter time change from unfertilized values was set at 5 min. The best fits (red line) for each parameter are shown **(A–D)** as compared to experimental data (black cross). The total amount of 4E-BP is expressed as % of initial value.

An 8-fold increase in k_off1_ leads to a 8-fold increase in the KD1/KD2 ratio (R1 and R2), such an increase would also be obtained through a k_on2_ 8-fold increase or a k_on1_ or k_off2_ 8-fold decrease. We therefore performed simulations with these different parameters, associated with the 32.5-fold increase in k_lysis4E−BP_ and a parameter time change of 5 min. The best fits (sum of square residual) for each parameter were respectively 1207 for k_off1_, 3692 for k_on2_, 3419 for k_on1_ and 4214 for k_off2_ (Figure 5) indicating that only the 8-fold increase in k_off1_ led to a good sum of square residual value.

**Figure 6:**
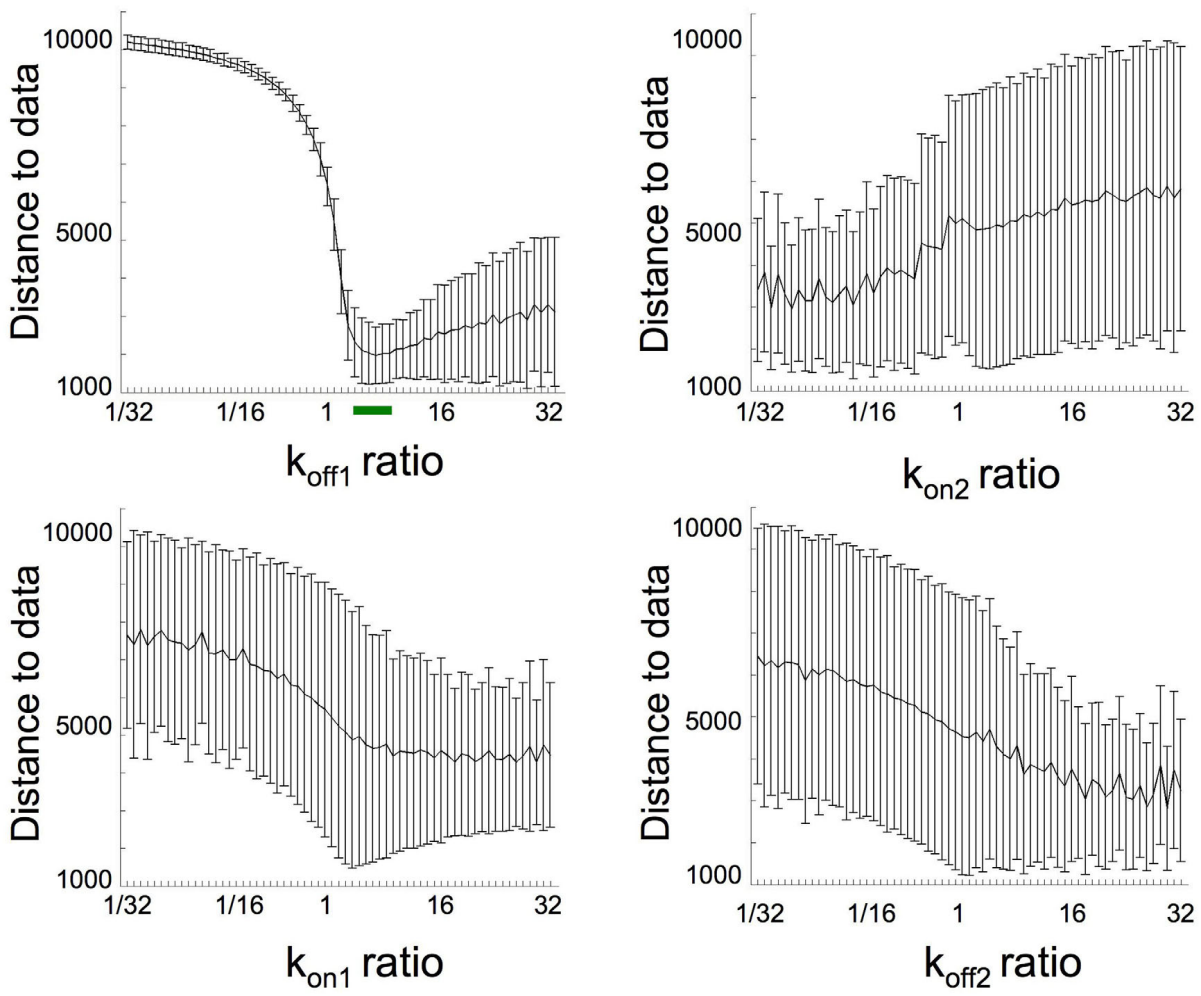
**Distance to data calculations from simulations performed varying together all of the parameters k_off1_, k_on2_, k_on1_, k_off2_**. Each indicated parameter varied by steps from 1/32 to 32-fold compared to unfertilized values in such a way that a 8-fold increase in KD_1_/KD_2_ was maintained constant (this gives 10434 possibilities). The k_lys4E-BP_ was fixed as 32.5-fold increase compared to unfertilized eggs and the parameter time change was fixed to 5 min. For each possible ratio (fertilized value vs. unfertilized value), the mean and *SD* of the distances to data were reported. Green bar corresponds to region where simulation fits well.

Figure 6 shows the distance to data (sum of square residual value and SD) calculated when each parameter varied by steps from 1/32 to 32-fold compared to unfertilized values. Simulation with the best fit (sum of square residual = 1189) was obtained with respectively a k_off1_ × 8, k_on1_ × 1, and k_on2_ and k_off2_ × 1/27. Regarding that this sum of square residual is not significantly different from the distance (1207) obtained with the unique k_off1_ variation, the k_off1_ role in eIF4E:4E-BP destabilization at fertilization appears to be the most relevant.

Altogether, the model allows to conclude that destabilization of eIF4E:4E-BP complex by an 8-fold increase of its dissociation rate (k_off1_) associated with a 32.5-fold increase in the mechanism of 4E-BP degradation, both taking place in 5 min following fertilization, are necessary and sufficient to explain the observed experimental changes occurring in the total amount of 4E-BP.

**Figure 7:**
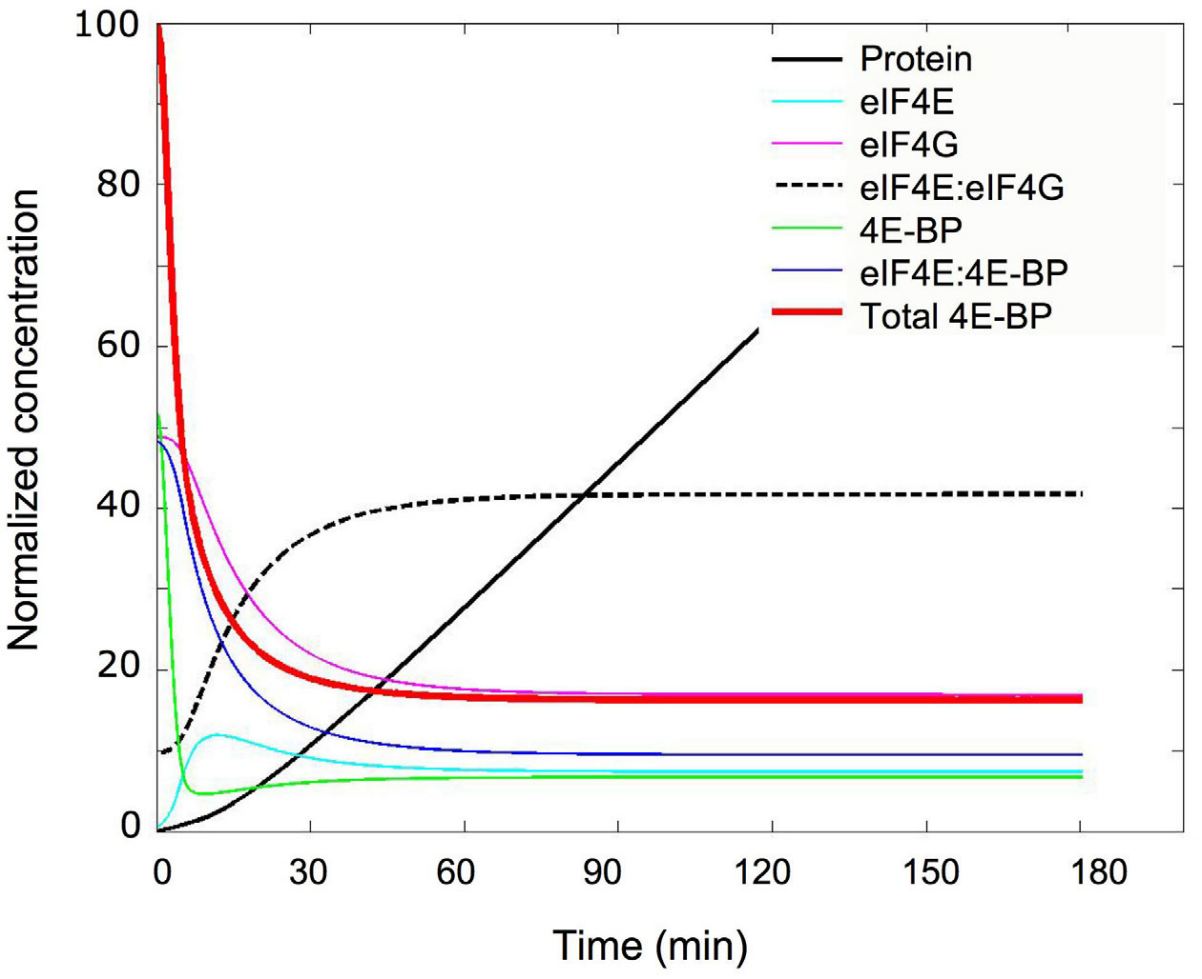
**Simulation of the concentration changes occurring at fertilization**. The concentration changes of the indicated constituents were calculated and plotted vs. time after fertilization using the parameters depicted in Table 1; “eIF4E,” “eIF4G,” and “4E-BP” are the concentrations of the free forms of these proteins; “eIF4E:eIF4G” and “eIF4E:4E-BP” correspond to the concentrations of the complexed forms; “Total 4E-BP” represents the total concentration of 4E-BP (free and complexed). Normalization was done with 100% corresponding to the initial concentration of total 4E-BP, i.e., 3.67 μM (see Table 1). The kinetic of accumulation of protein was calculated from the kinetic of eIF4E:4E-BP changes and is shown in relative units (black curve).

Using the minimal model, the parameters determined in the unfertilized eggs and the changes predicted in the fertilized eggs (Table 1) the kinetic of concentration changes of all parameters were simulated (Figure 7). Importantly, the concentration of eIF4E:eIF4G complex increased 4.2-fold from its initial value in the unfertilized eggs, in a time interval of 30 min. The resulting calculated increase and the protein accumulation kinetic (Figure 7, black line) were highly compatible with the *in vivo* protein synthesis changes after fertilization (see Figure 3B). The 4.2-fold increase in eIF4E:eIF4G complex compares favorably with the experimental 6.6-fold increase in protein synthesis measure as described in the material and methods section (*SD* = 2.3, *n* = 8 independent experiments). Although we are aware that reaction R4 of the model is a very highly simplified representation of the mechanism of cap-dependent translation, and that many other changes on translation factors have been reported at fertilization in sea urchin (Oulhen et al., 2007; Belle et al., 2011; Costache et al., 2012) the assumption that translation was proportional to the amount of eIF4E:eIF4G complex appeared rational in the minimal model elaboration.

### Simulation of rapamycin effect on 4E-BP changes and protein synthesis increase at fertilization

It has been already reported that FRAP/mTOR is involved in the protein synthesis increase following fertilization (Salaun et al., 2003, 2004, 2005; Oulhen et al., 2007). The effect of rapamycin, an inhibitor of the protein kinase FRAP/mTOR, has been analyzed in early development of sea urchin embryo. Protein synthesis measured as described in the Materials and Methods section was 53.0% (*SD* = 5.1, *n* = 8) of the value in control fertilized eggs. Rapamycin slowed down the decrease in the total amount 4E-BP, which stabilized at 41.7% (*SD* = 5.4, *n* = 8) of unfertilized value after 30 min. This observation was rather surprising since this value is higher than the value in fertilized eggs (18%), while protein synthesis was decreased as compared to control fertilized eggs.

**Figure 8:**
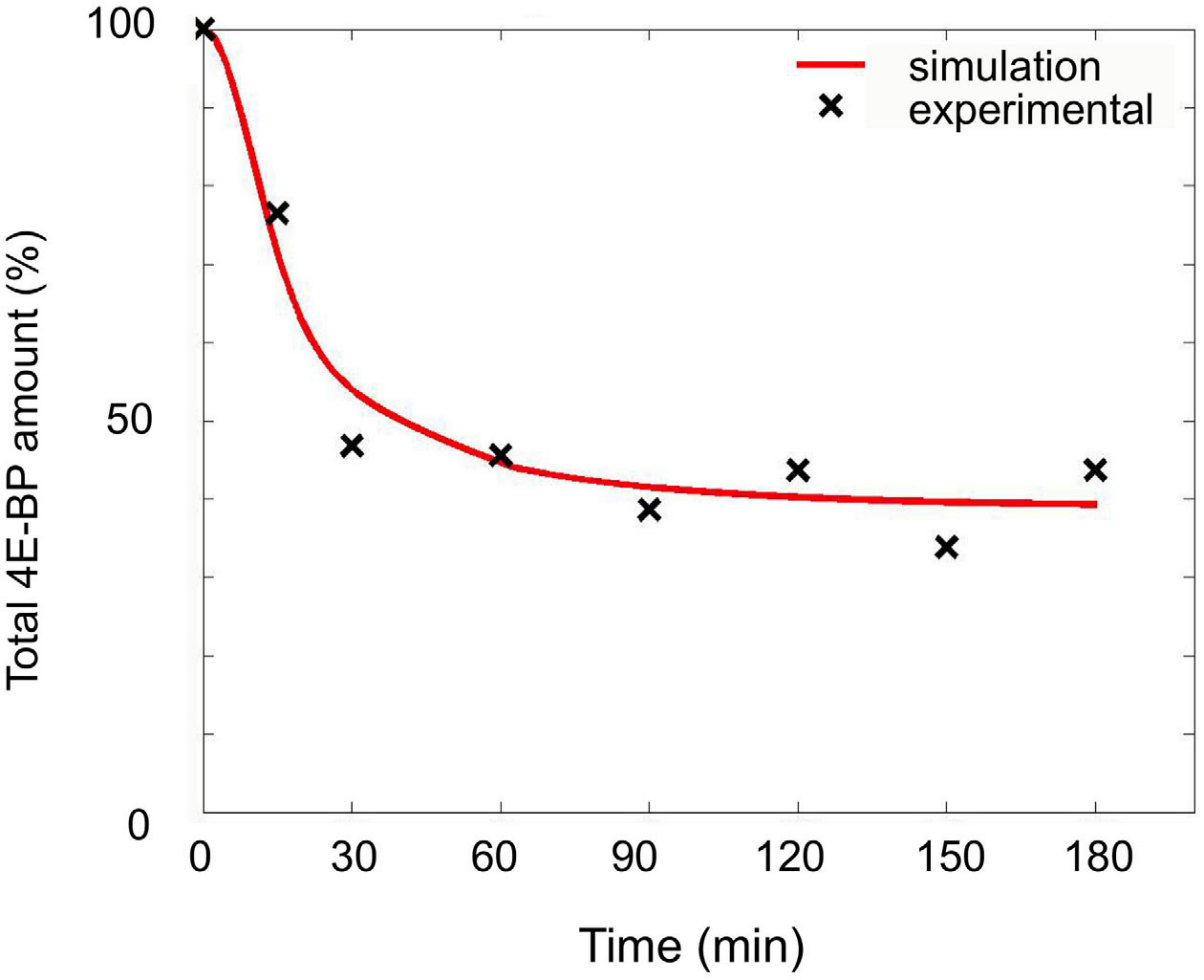
**Simulation of fertilization changes on 4E-BP in the presence of rapamycin**. Simulated curve (red line) obtained with optimized parameters (see text) compared to experimental data (black cross). The total amount of 4E-BP is expressed as % of initial value.

The changes observed in the presence of rapamycin were modeled as a modification of the parameters of the minimal model. Simulations were performed with k_off1_ ranging from 1 to 8-fold, k_lysis4E−BP_ from 1 to 32.5-fold and changes for time parameter from 1 to 100 min. A good fit was obtained for a k_off1_ 1-fold the value in unfertilized eggs, k_lysis4E−BP_ value 16-fold the unfertilized value and a parameter time change of 33 min (Figure 8 and Table 1). In these conditions, the concentration of eIF4E:eIF4G at steady state was 2.58-fold compared to the 4.25-fold in fertilized eggs in the absence of rapamycin. The model therefore predicts a rapamycin-induced limited increase in protein synthesis of 53.6%, in very good concordance with the experimental data value of 52.0%.

Therefore the model predicted that not only k_off1_ increase after fertilization but also the degradation mechanism of 4E-BP is under the control of FRAP/mTOR pathway.

## Discussion

While the regulation of protein synthesis is a highly complex process, we show here that using a minimal model based on three main actors eIF4E, eIF4G, and 4E-BP, the changes occurring at fertilization in sea urchin could be fully simulated with adjustment of a minimum of parameters. The model postulates that fertilization triggers two events: (1) an increase in the KD_1_ of the eIF4E:4E-BP complex due to an 8-fold increase of the dissociation constant (k_off1_) and (2) a 32.5-fold increase in the mechanism of 4E-BP degradation (k_lysis4E−BP_). Therefore, the disappearance of 4E-BP cannot result from the unique dissociation of the eIF4E:4E-BP complex. Interestingly, in mammalian cells it has been found that 4E-BP1 is degraded in eIF4E-knock-down cells (Yanagiya et al., 2012). The authors show that the non-eIF4E-bound hyperphosphorylated 4E-BP1 is degraded while eIF4E-bound hypophosphorylated 4E-BP1 is stable reinforcing our assumption in the model that only free 4E-BP is degraded. However, it should be noted that there is no indication in this study of an activation of the mechanism of degradation itself while it is the case in sea urchin fertilization.

As expected, the model predicts that rapamycin reverses the fertilization-induced change in k_off1_. An attractive hypothesis would be that fertilization in sea urchin activates the kinase FRAP/mTOR that phosphorylates 4E-BP and increases the k_off1_ of its association with eIF4E, resulting in a destabilization of eIF4E:4E-BP complex. A role for FRAP/mTOR protein kinase on the destabilization of eIF4E:4E-BP complex at fertilization is compatible with the observations (1) that rapamycin, a FRAP/mTOR inhibitor, inhibits protein synthesis increase in sea urchin fertilization, and inhibits the disappearance of 4E-BP upon fertilization and (2) that the 4E-BP protein was shown to be a substrate for FRAP/mTOR in other organisms; in mammals, multiple and hierarchical phosphorylation events provoke the release of 4E-BP1 from eIF4E, in concordance with a KD_1_ increase (Gingras et al., 2001). Although the relationship between FRAP/mTOR activity and the affinity of 4E-BP for eIF4E is not established in sea urchin embryos (Oulhen et al., 2009), it has been shown that the degradation of 4E-BP correlates with its phosphorylation (Cormier et al., 2001). Therefore, the possibility that fertilization activates FRAP/mTOR resulting in a k_off1_ increase should be experimentally analyzed when the purified proteins will be available under their non-phosphorylated and phosphorylated forms.

We cannot exclude that fertilization could have also modified KD_2_. Since eIF4G undergoes electrophoretic changes at fertilization (Oulhen et al., 2007) it would be of interest to identify the post-translational changes occurring on this factor, and its consequences on eIF4E:eIF4G interaction.

Furthermore and unexpectedly, the model also predicts that the mechanism of 4E-BP degradation was also rapamycin-sensitive. Very interestingly, the degradation mechanism of 4E-BP appears to be a FRAP/mTOR target, which could also be biochemically tested when the mechanism will be identified. For now, this feature is described for the first time using sea urchin fertilization and would be of great interest to be searched in other species and other physiological regulations.

## Author contributions

Sébastien Laurent and Adrien Richard equally contributed to the article as first author. Sébastien Laurent and Didier Flament designed and realized the SPR experiments. Virginie Glippa determined 4E-BP concentration in sea urchin eggs. Pauline Gosselin prepared and provided the proteins for SPR determinations. Odile Mulner-Lorillon, Julia Morales, Patrick Cormier provided the biochemical data on translation activity and protein accumulation. and Robert Bellé designed and coordinated the study. All authors contributed to the model elaboration and to the analysis of the results.

### Conflict of interest statement

The authors declare that the research was conducted in the absence of any commercial or financial relationships that could be construed as a potential conflict of interest. The Guest Associate Editor Thierry Tonon declares that, despite being affiliated to the same institution as authors Patrick Cormier, Virginie Glippa, Julia Morales and Odile Mulner-Lorillon, the review process was handled objectively and no conflict of interest exists.

## References

[B1] Abiko F. Tomoo K. Mizuno A. Morino S. Imataka H. Ishida T. (2007). Binding preference of eIF4E for 4E-binding protein isoform and function of eIF4E N-terminal flexible region for interaction, studied by SPR analysis. Biochem. Biophys. Res. Commun. 355, 667–672 10.1016/j.bbrc.2007.01.198 17316564

[B2] Belle R. Pluchon P. F. Cormier P. Mulner-Lorillon O. (2011). Identification of a new isoform of eEF2 whose phosphorylation is required for completion of cell division in sea urchin embryos. Dev. Biol. 350, 476–483 10.1016/j.ydbio.2010.12.015 21167828

[B3] Belle R. Prigent S. Siegel A. Cormier P. (2010). Model of cap-dependent translation initiation in sea urchin: a step towards the eukaryotic translation regulation network. Mol. Reprod. Dev. 77, 257–264 10.1002/mrd.21142 20014323

[B4] Brunn G. J. Fadden P. Haystead T. A. Lawrence J. C. Jr. (1997). The mammalian target of rapamycin phosphorylates sites having a (Ser/Thr)-Pro motif and is activated by antibodies to a region near its COOH terminus. J. Biol. Chem. 272, 32547–32550 10.1074/jbc.272.51.32547 9405468

[B5] Burnett P. E. Barrow R. K. Cohen N. A. Snyder S. H. Sabatini D. M. (1998). RAFT1 phosphorylation of the translational regulators p70 S6 kinase and 4E-BP1. Proc. Natl. Acad. Sci. U.S.A. 95, 1432–1437 10.1073/pnas.95.4.1432 9465032 PMC19032

[B6] Calzone L. Fages F. Soliman S. (2006). BIOCHAM: an environment for modeling biological systems and formalizing experimental knowledge. Bioinformatics 22, 1805–1807 10.1093/bioinformatics/btl172 16672256

[B7] Cormier P. Pyronnet S. Morales J. Mulner-Lorillon O. Sonenberg N. Belle R. (2001). eIF4E association with 4E-BP decreases rapidly following fertilization in sea urchin. Dev. Biol. 232, 275–283 10.1006/dbio.2001.0206 11401391

[B8] Cormier P. Pyronnet S. Salaun P. Mulner-Lorillon O. Sonenberg N. (2003). Cap-dependent translation and control of the cell cycle. Prog. Cell Cycle Res. 5, 469–475 14593742

[B9] Costache V. Bilotto S. Laguerre L. Belle R. Cosson B. Cormier P. (2012). Dephosphorylation of eIF2alpha is essential for protein synthesis increase and cell cycle progression after sea urchin fertilization. Dev. Biol. 365, 303–309 10.1016/j.ydbio.2012.03.002 22425618

[B10] Epel D. (1990). The initiation of development at fertilization. Cell Differ. Dev. 29, 1–12 10.1016/0922-3371(90)90019-S 2154300

[B11] Gilbert S. F. (2003). Developmental Biology. Sunderland, MA: Sinauer Associates

[B12] Gingras A. C. Gygi S. P. Raught B. Polakiewicz R. D. Abraham R. T. Hoekstra M. F. (1999b). Regulation of 4E-BP1 phosphorylation: a novel two-step mechanism. Genes Dev. 13, 1422–1437 10.1101/gad.13.11.1422 10364159 PMC316780

[B13] Gingras A. C. Raught B. Sonenberg N. (1999a). eIF4 initiation factors: effectors of mRNA recruitment to ribosomes and regulators of translation. Annu. Rev. Biochem. 68, 913–963 10.1146/annurev.biochem.68.1.913 10872469

[B14] Gingras A. C. Raught B. Sonenberg N. (2001). Regulation of translation initiation by FRAP/mTOR. Genes Dev. 15, 807–826 10.1101/gad.887201 11297505

[B15] Gosselin P. Oulhen N. Jam M. Ronzca J. Cormier P. Czjzek M. (2011). The translational repressor 4E-BP called to order by eIF4E: new structural insights by SAXS. Nucleic Acids Res. 39, 3496–3503 10.1093/nar/gkq1306 21183464 PMC3082885

[B16] Haghighat A. Mader S. Pause A. Sonenberg N. (1995). Repression of cap-dependent translation by 4E-binding protein 1: competition with p220 for binding to eukaryotic initiation factor-4E. EMBO J. 14, 5701–5709 8521827 10.1002/j.1460-2075.1995.tb00257.x PMC394685

[B17] Hinnebusch A. G. (2011). Molecular mechanism of scanning and start codon selection in eukaryotes. Microbiol. Mol. Biol. Rev. 75, 434–467 10.1128/MMBR.00008-11 21885680 PMC3165540

[B18] Jackson R. J. Hellen C. U. Pestova T. V. (2010). The mechanism of eukaryotic translation initiation and principles of its regulation. Nat. Rev. Mol. Cell Biol. 11, 113–127 10.1038/nrm2838 20094052 PMC4461372

[B19] Jackson R. J. Hellen C. U. Pestova T. V. (2012). Termination and post-termination events in eukaryotic translation. Adv. Protein Chem. Struct. Biol. 86, 45–93 10.1016/B978-0-12-386497-0.00002-5 22243581

[B20] Jackson R. J. Hunt S. L. Reynolds J. E. Kaminski A. (1995). Cap-dependent and cap-independent translation: operational distinctions and mechanistic interpretations. Curr. Top. Microbiol. Immunol. 203, 1–29 10.1007/978-3-642-79663-0_1 7555086

[B21] Joshi B. Lee K. Maeder D. L. Jagus R. (2005). Phylogenetic analysis of eIF4E-family members. BMC Evol. Biol. 5:48 10.1186/1471-2148-5-48 16191198 PMC1260017

[B22] Mader S. Lee H. Pause A. Sonenberg N. (1995). The translation initiation factor eIF-4E binds to a common motif shared by the translation factor eIF-4 gamma and the translational repressors 4E-binding proteins. Mol. Cell. Biol. 15, 4990–4997 7651417 10.1128/mcb.15.9.4990 PMC230746

[B23] Mathews M. B. Sonenberg N. Hershey J. W. B. (2007). Origins and principles of translational control, in Translational Control in Biology and Medecine, eds Sonenberg N. Hershey J. Mathews M. (New York, NY: CSHL Press), 1–31

[B24] Mizuno A. In Y. Fujita Y. Abiko F. Miyagawa H. Kitamura K. (2008). Importance of C-terminal flexible region of 4E-binding protein in binding with eukaryotic initiation factor 4E. FEBS Lett. 582, 3439–3444 10.1016/j.febslet.2008.09.003 18789325

[B25] Morales J. Mulner-Lorillon O. Cosson B. Morin E. Belle R. Bradham C. A. (2006). Translational control genes in the sea urchin genome. Dev. Biol. 300, 293–307 10.1016/j.ydbio.2006.07.036 16959243

[B26] Oulhen N. Boulben S. Bidinosti M. Morales J. Cormier P. Cosson B. (2009). A variant mimicking hyperphosphorylated 4E-BP inhibits protein synthesis in a sea urchin cell-free, cap-dependent translation system. PLoS ONE 4:e5070 10.1371/journal.pone.0005070 19333389 PMC2659438

[B27] Oulhen N. Mulner-Lorillon O. Cormier P. (2010). eIF4E-binding proteins are differentially modified after ammonia versus intracellular calcium activation of sea urchin unfertilized eggs. Mol. Reprod. Dev. 77, 83–91 10.1002/mrd.21110 19777548

[B28] Oulhen N. Salaun P. Cosson B. Cormier P. Morales J. (2007). After fertilization of sea urchin eggs, eIF4G is post-translationally modified and associated with the cap-binding protein eIF4E. J. Cell Sci. 120, 425–434 10.1242/jcs.03339 17213333

[B29] Parrington J. Davis L. C. Galione A. Wessel G. (2007). Flipping the switch: how a sperm activates the egg at fertilization. Dev. Dyn. 236, 2027–2038 10.1002/dvdy.21255 17654712

[B30] Pause A. Belsham G. J. Gingras A. C. Donze O. Lin T. A. Lawrence J. C. (1994). Insulin-dependent stimulation of protein synthesis by phosphorylation of a regulator of 5'-cap function. Nature 371, 762–767 10.1038/371762a0 7935836

[B31] Pyronnet S. Imataka H. Gingras A. C. Fukunaga R. Hunter T. Sonenberg N. (1999). Human eukaryotic translation initiation factor 4G (eIF4G) recruits mnk1 to phosphorylate eIF4E. EMBO J. 18, 270–279 10.1093/emboj/18.1.270 9878069 PMC1171121

[B32] Rousseau D. Gingras A. C. Pause A. Sonenberg N. (1996). The eIF4E-binding proteins 1 and 2 are negative regulators of cell growth. Oncogene 13, 2415–2420 8957083

[B33] Salaun P. Boulben S. Mulner-Lorillon O. Belle R. Sonenberg N. Morales J. (2005). Embryonic-stage-dependent changes in the level of eIF4E-binding proteins during early development of sea urchin embryos. J. Cell Sci. 118, 1385–1394 10.1242/jcs.01716 15769855

[B34] Salaun P. Le Breton M. Morales J. Belle R. Boulben S. Mulner-Lorillon O. (2004). Signal transduction pathways that contribute to CDK1/cyclin B activation during the first mitotic division in sea urchin embryos. Exp. Cell Res. 296, 347–357 10.1016/j.yexcr.2004.02.013 15149864

[B35] Salaun P. Pyronnet S. Morales J. Mulner-Lorillon O. Belle R. Sonenberg N. (2003). eIF4E/4E-BP dissociation and 4E-BP degradation in the first mitotic division of the sea urchin embryo. Dev. Biol. 255, 428–439 10.1016/S0012-1606(02)00099-4 12648502

[B36] Sonenberg N. Gingras A. C. (1998). The mRNA 5' cap-binding protein eIF4E and control of cell growth. Curr. Opin. Cell Biol. 10, 268–275 10.1016/S0955-0674(98)80150-6 9561852

[B37] Umenaga Y. Paku K. S. In Y. Ishida T. Tomoo K. (2011). Identification and function of the second eIF4E-binding region in N-terminal domain of eIF4G: comparison with eIF4E-binding protein. Biochem. Biophys. Res. Commun. 414, 462–467 10.1016/j.bbrc.2011.09.084 21964297

[B38] Von Der Haar T. Oku Y. Ptushkina M. Moerke N. Wagner G. Gross J. D. (2006). Folding transitions during assembly of the eukaryotic mRNA cap-binding complex. J. Mol. Biol. 356, 982–992 10.1016/j.jmb.2005.12.034 16405910

[B39] Yanagiya A. Suyama E. Adachi H. Svitkin Y. V. Aza-Blanc P. Imataka H. (2012). Translational homeostasis via the mRNA cap-binding protein, eIF4E. Mol. Cell 46, 847–858 10.1016/j.molcel.2012.04.004 22578813 PMC4085128

